# Strategic Human Resource Management and COVID‐19: Emerging Challenges and Research Opportunities

**DOI:** 10.1111/joms.12695

**Published:** 2021-03-18

**Authors:** David G. Collings, John McMackin, Anthony J. Nyberg, Patrick M. Wright

**Affiliations:** ^1^ Dublin City University; ^2^ University of South Carolina

**Keywords:** business leadership, COVID‐19, stakeholder perspective, strategic human resource management, work

At its core the COVID‐19 pandemic is a human crisis. Thus, human resource (HR) leaders have been central to the response in organisations globally. This contrasts with previous crises such as the global recession of 2008–09 or the Y2K crisis at the turn of the millennium that accentuated the roles of finance and IT leaders, respectively. By amplifying the role of HR leaders, COVID‐19 has become an inflection point with substantive implications for HR globally. In this commentary, we reflect on the implications of COVID‐19 for HR research, including identifying some key research questions for strategic human resource management (HRM).

Early in the evolution of the field, Wright and McMahan defined strategic HRM as ‘the pattern of planned human resource deployments and activities intended to enable the firm to achieve its goals’ (1992, p. 298). They argued that the domain of strategic HRM encompassed ‘the determinants of decisions about HR practices, the composition of human capital resource pools, the specification of the required human resource behaviours, and the effectiveness of these decisions given various business strategies and/or competitive situations’ (Wright and McMahon, 1992, p. 298). Since then, strategic HRM research has overwhelmingly focused on the relationship between HR practices and firm performance (Huselid, 1995) or the impact of those practices on mediators between these two variables (Boxall et al., 2016). However, the global pandemic revealed how myopic such research has been for addressing how firms strategically manage people.

This narrow focus limits our understanding of key questions exposed during COVID‐19. For example, the pandemic requires a shift in understanding of how work context, such as working onsite versus working from home (WFH), influences employee behaviours and actions. The pandemic has also exposed tensions among stakeholders and challenges the primacy of the shareholder view which has dominated thinking in strategic HRM (Crane and Matten, 2020; Hitt et al., 2020). Finally, while HR has taken a strategic turn, tensions around its role have been exposed during the pandemic, highlighting the requirement for both strategic and tactical contributions. We consider each of these in turn.

## THE IMPACT OF WORK CONTEXT

COVID‐19 has changed the experience of work for the vast majority of employees. It forced organisations across the globe to adapt how work is organised and how jobs are designed. The potential for fractures between employee groups has also increased. For example between those who can WFH and those who cannot, those who remained on payroll versus those furloughed, and even those in different business units impacted differently by the pandemic.

Strategic HRM research has been critiqued for its failure, or conceptual inability, to include novel, contemporary HR practices (Harney and Collings, 2021). Thus, traditional conceptualisations of HR practices or high‐performance work systems require modification in the context of COVID‐19. Strategic HRM research needs to move toward the more nuanced conceptualisation and measurement of HR practices such as flexibility, job design etc. rather than capturing these under the category of ‘other’ which has been far too common in extant research (Boon et al., 2019).

Indeed, academic research in strategic HRM is often premised on a homogenous view of the employee relationship and an assumption that HR practices used within firms are relatively homogenous (c.f. Huselid, 1995). However, employees differ in their experience and interpretation of HR practices depending on their role (Kehoe and Wright, 2013). Lepak and Snell (1999) were among the first to differentiate strategic HRM practices for different employee groups. While such a differentiated approach has been core to discussions on talent management (Collings et al., 2019) we argue that COVID‐19 highlights the importance of considering the differential impact of strategic HRM across different employee groups in terms of how and where they work.

These changes also challenge many assumptions underpinning, and conclusions drawn from, traditional strategic HRM research. For example, this research generally assumes workers are located in a physical workplace, with limited consideration of managing a virtual workforce. Where HR flexibility has been considered, this has largely focused on HR practices, with culture/values overlooked. Understanding how culture and organisational values influence how place of work impacts on outcomes such as individual or unit performance remain key research questions. Likewise, on the rare occasions that strategic HRM research considers communications, this is generally limited to communicating financial information at the firm level. How communication differentiated those organisations that responded most effectively to the pandemic versus those that did not could provide insight into how and why some organizations and leaders respond better to crises than others.

## TOWARDS A STAKEHOLDER PERSPECTIVE

Strategic HRM research has overwhelmingly focused on shareholder value as a key outcome and the implications of such a narrow focus have been exposed by the COVID‐19 pandemic (see also Crane and Matten, 2020). Beer and colleagues' (1985) seminal framework did incorporate multiple stakeholders, including shareholders, employees, trade unions, management, and government. They also considered a broader range of outcomes including economic value, individual wellbeing, and societal benefits. However, these perspectives are rare in strategic HRM literature (c.f. Beer et al., 2015; Guest et al., 2012). COVID‐19 has however elevated the consideration of employees as critical stakeholders. By its essence COVID‐19 is a threat to the health and safety of employees requiring organisations to evaluate employee risk. For instance, some employees were harmed by firms’ continued narrow focus on short‐term financial outcomes while failing to balance employee needs (Collings et al., 2021).

The pandemic also highlighted the importance of customers as stakeholders as organisations – something often taken for granted in management research but rarely in HR research (Ulrich et al., 2012). Customers remain one of the least studied stakeholders in strategic HRM research despite calls for their consideration (Beer et al., 2015). Indeed, firms need to carefully manage the trade‐offs between employees, customers, and shareholders. Through the pandemic employees needed to work because customers need products or services, and firms also had to manage customer and employee safety that at times during the pandemic came at the expense of shareholder returns. Two examples of research focused on customers include examining HR practices targeted at enhancing customer service, and the relationship to firm outcomes such as service quality (Liao et al., 2009), and research showing that HR practices that influence how employees treat customers also affects how customers treat employees influencing employee satisfaction and turnover (Shepherd et al., 2020). Future research needs to consider how HR practices need to be modified to account for shifts in service delivery, such as increased digitisation of service provision or reduced physical interaction in service provision, in response to COVID‐19 and how these impact key customer outcomes. Research could also consider how customer perceptions of how employees have been managed in response to COVID‐19 impacts on their trust in the products and services of those firms and how they engage with them.

Strategic HRM research should also recognize the role of communities as important stakeholders. COVID‐19 has burdened many communities around the world. When organizations go beyond exclusively focusing on financial stakeholders and commit to the local community, they can make substantive, long‐term positive differences in ways that governments cannot. This is an area where research that bridges strategic HRM and corporate social responsibility is particularly welcome (see Crane and Matten, 2020). For example, how have corporate volunteering efforts, mitigated the impact of COVID‐19 on local communities? Likewise, organisations are at the forefront of influencing employee (and consequently community) vaccination efforts and inclusion conversations, and research should examine how strategic HRM practices influence employee and community behaviours, and how the cumulated effects influence organisational outcomes.

Overall, the challenges associated with COVID‐19 highlight for HR practitioners, and should for strategic HRM researchers, the need to balance multiple stakeholders needs (Collings et al., 2021). It is notable that this awakening in strategic HRM parallels recent calls in strategy research to expand the stakeholder perspective (Hitt et al., 2020).

## INTEGRATING STRATEGIC AND TACTICAL RESPONSES

As HR research and practices evolve towards a more strategic orientation the conflict between the traditional employee centred or welfare capitalist roles of HR and its incarnation as strategic business partner has become a point of tension (Prichard, 2010). Although the strategic business partner model has been core to the strategic positioning of the HR function (Ulrich, 1996), the oversimplified implementation of this model has been critiqued as a separation of ‘thinking from doing’, thereby creating an artificial boundary between strategic and transactional work (Reilly et al., 2007). Strategic HR work has become highly valued, while transactional or tactical HR work devalued, often delegated to line managers, or concentrated in centres of excellence (Prichard, 2010). This is reflected in a shift from evaluating HR effectiveness against technical criteria established by the profession (e.g., validity) to evalutations of other stakeholders (Beer et al., 2015).

COVID‐19 highlights the need to recalibrate the discussion on the tactical versus strategic role of strategic HRM. For example, how has the HR function navigated tensions in their role as employee advocate and strategic business partner? Extant research has highlighted the positive impact of HR’s recognized unique operational competencies, such as short‐term retrenchment measures, as opposed to some perceived strategic orientation, in aiding organisations to navigate the great recession (Roche and Teague, 2012). However, a solely operational focus cannot generate sustainable outcomes, suggesting that the most effective leaders will balance this tactical role with a strategic influence.

Strategic HRM research has also been criticised for drawing on ‘narrow and classical definitions of strategy implying pre‐determined consensus and a linear sequential progress from formulation through implication’ implying that HR strategy is developed as a once off structural intervention (Harney and Collings, 2021, p. 3). The pace of change during the pandemic necessitated a dynamic and fluid approach to strategy. Thus, COVID‐19 provides an opportune context for research which explores the strategizing process in executive leadership teams and how HR strategy has emerged and evolved in that context.

## CONCLUSIONS

We highlight the central role that HR is playing driving operational and strategic success during the COVID‐19 pandemic. We illuminate three substantive implications for strategic HRM research. First, the pandemic highlights a need to expand understanding about how work context influences employee behaviours and actions. Second, it exposes tensions among stakeholders, highlighting the need to consider *inter alia* employees, customers, and communities along with shareholders. Third, tensions between the strategic and operational roles of HR are exposed. In this commentary, we reflect on the implications of COVID‐19 for research on strategic HRM and identify key research questions for future scholarship.
